# "Ierne" Taken to Task

**Published:** 1918-10-12

**Authors:** 


**THE EDITOR'S LETTER-BOX—(*continued*).**

### "Ierne" Taken to Task.

### A Sister's Lecture, with "Ierne's" Five Questions.

*To the Editor of* The Hospital.

Sir,—Having perused the majority of "Ierne's" articles, I have come to the conclusion that it is time that someone should endeavour to counteract their baneful influence.

I give "Ierne" credit for the best of motives, but, in his (?) ignorance of the tenets and ideals of the nursing profession, he is engineering an agitation which shows plainly that he does not know the meaning of the word "loyalty." The suggestion that we should band ourselves together in a sort of trade union, and voice our grievances after the manner of Socialist agitators or discontented employees, is absolutely derogatory to the profession. Nurses are members of one of the noblest of professions, and, moreover, our profession is also a vocation—a fact which has apparently never occurred to "Ierne." That there are many reforms needed no one denies, the heads of our profession least of all—and they are energetically setting their shoulders to the task of finding the best way in which to make improvements.

We nurses can confidently await the help of our College to remedy existing evils, and we do not need to "agitate" at all. Nurses, at the various meetings and conferences, have had ample means of putting forward any point of view—the most recently certificated nurse has been free to say as much as the matron of ten years' standing.

"Ierne" is striving to promote a spirit of unrest among the rank and file of the nursing profession. I deplore the influence he (?) may possibly have upon young nurses who do not perhaps as yet sufficiently love their calling to realise its sacredness. The art of the healing of the sick is a beautiful work; shall we debase it by selfish aims, by agitating for this or that privilege as if we were women working solely for gain? I do not wish to shelve the practical side; the profession is also the sole means of livelihood to many of its members, but let nurses remember that this is by no means forgotten by those who have its best interests at heart. Matrons are the first to acknowledge that some of the conditions of employment might be improved. Every rise of salary, every extra off-duty time has invariably been granted upon the representation of the matron. It is she who takes the trouble to make the nurses' quarters as homelike as possible. Many reforms would have been effected years ago had the matron a free hand, which she has not. Moreover, let those nurses (and others) who glibly talk of "an eight-hour day," "a day off a week," etc., just try to work out a time-table of the off-duty time in their own ward, and see if they do not speedily give it up in despair! What, then, is the matron's task, who has to apportion the days off, etc., for the whole hospital in such a manner that there be a sufficient number of senior and junior nurses on duty in each ward to ensure the efficient carrying-on of the work.

Let nurses cleave to their ideals; let them work contentedly for the relief of the sick. Let them not worry about those unfavourable economic conditions which will before very long be remedied by those who have been elected by the nurses for this very task, among others.

The spirit of the age is, I suppose, responsible for the fact that one can read such "agitations" as have lately appeared in our nursing papers. A nurse may be both broad-minded and progressive—but nevertheless resent that an outsider (which "Ierne" obviously is) should take upon himself (?) to stir up discontent among the rank and file of the profession to which she is proud to belong. Nurses, let us be loyal, in the best and widest interpretation of the word, to our profession, to our matrons, and to each other. Let us older ones also, while working for the realisation of our noblest ideals, give a helping hand to the probationers and lead them to see the great beauty of the true ideals of the nursing profession.—Yours faithfully,

F. N.

[I should like to help this correspondent to clearer vision. Abuse or defence of "Ierne" counts for nothing, and I am void of feeling as to being described as ignorant, disloyal, an outsider, a Socialist agitator, or what you please. Abuse is waste of time, energy, and in this case of paper. May I invite my critic to send a second letter, and to answer the following questions? (1) Who deprives the matron of "a free hand"? (2) Is such an "outsider," or "disloyal"? (3) Do the British public, in the writer's opinion, expect that their sick poor shall be nursed "on the cheap" by women who have a nursing vocation? (4) Do "the tenets and ideals of the nursing profession" provide that in no circumstances shall nurses articulate or complain? (5) Is it necessarily disloyalty to high ideals that seeks to raise the status of the profession so that the best, rather than the second best, class of recruits shall seek entrance? Let us come down to facts and hang up the halo argument.—Ierne.]

